# Pulmonary melanoma and “crazy paving” patterns in chest images: a case report and literature review

**DOI:** 10.1186/s12885-016-2630-5

**Published:** 2016-08-03

**Authors:** Yikuan Feng, Jianping Zhao, Qun Yang, Weining Xiong, Guohua Zhen, Yongjian Xu, Zhenxiang Zhang, Huilan Zhang

**Affiliations:** Department of Respiratory and Critical Care Medicine, Tongji Hospital, Tongji Medical College, Huazhong University of Science and Technology, Wuhan, 430030 China

**Keywords:** Pulmonary, Melanoma, Crazy paving, Case report

## Abstract

**Background:**

In the lung, melanoma is mostly arranged as patterns of multiple nodules, solitary nodules, or miliary invasions. Very rarely, it also displays a “crazy paving” pattern (also described as a “paving stone,” “flagstone,” or “slabstone” pattern), which is rarer still in discrete bilateral nodules. This pattern is considered to be caused by pulmonary alveolar proteinosis, but its association with various diseases is unclear.

**Case presentation:**

A 60-year-old man was diagnosed with pulmonary melanoma. Computed tomography revealed discrete bilateral nodules surrounded by a “paving” pattern. A literature review found more than 40 types of diseases that have presented with “paving” patterns in the lung—predominantly pulmonary alveolar proteinosis, viral pneumonia, exogenous lipoid pneumonia, bacterial pneumonia, pulmonary alveolar microlithiasis, interstitial pneumonia, ARDS, squalene aspiration pneumonia, radiation pneumonitis, drug-induced pneumonitis, pulmonary leptospirosis, pulmonary hemorrhage, and pulmonary nocardiosis.

**Conclusions:**

We describe the first case of pulmonary melanoma in the form of discrete bilateral nodules accompanied with a computed tomography paving pattern. Although pulmonary paving patterns are rare, more than 40 diseases reportedly display them; clinicians should consider melanoma of the lung in differential diagnoses for patients who show such a pattern.

## Background

Melanoma accounts for approximately 75 % of deaths from skin cancers, and has an increasing incidence rate [1, 2]. Although it can invade all organs, the lung is the most frequently involved, with a 70–87 % incidence rate of metastatic invasion [3, 4]. Melanoma in the lung is mostly metastatic, and usually forms patterns of multiple nodules, solitary nodules, or miliary invasions. Diffuse pulmonary infiltration together with discrete bilateral nodules is an exceedingly scarce pattern. Here, we describe the first case, to our knowledge, of melanoma infiltrating the lung in the pattern of bilateral discrete nodules accompanied with surrounding “crazy paving” lesions in computed tomography (CT) images. We also review the literature on this imaging pattern (also described as a “paving stone,” “flagstone,” and “slabstone” pattern) and its association with various diseases.

## Case presentation

A 60-year-old Asian man with a smoking history of more than 120 pack-years presented to our department with complaints of intermittent dry cough, hemoptysis, suppression of chest, and dyspnea for 3 months. He denied fever, weakness, or weight loss. Two months before admission to our department, the patient received a 2-week course of antibiotic therapy that showed no effectiveness.

On presentation, the patient was a well-nourished man with temperature of 36.5 °C, blood pressure of 105/60 mmHg, pulse rate of 65 beats/min, respiratory rate of 19 breaths/min, and oxygen saturation of 92 % on ambient air. Arterial blood gas analysis showed PaCO_2_ 43 mmHg, PaO2 66 mmHg, and SaO2 92 %. Auscultation of his chest revealed decreased breath sounds and fine crackles bilaterally, but more notably in the left lower lung. He showed no evidence of suspicious pigmented lesions of the skin, mucosa, or eyes; the rest of the physical examination was unremarkable.

His complete blood count showed a mild anemia level of hemoglobin 9.8 g/dL and mild leukocytosis with a white blood cell count of 10.46 × 109/L and neutrophils at 7.72 × 109/L. Metabolic panel was normal except for a serum potassium level of 2.89 mmol/L. Serum tumor markers, including CEA, SCC, CYFRA21-1, NSE, AFP, CA19-9, and CA72-4, were all normal. Serum antinuclear antibody test, anti-neutrophil cytoplasmic antibody test, thyroid function test, erythrocyte sedimentation rate, and T-SPOT were also all negative, as were tests for hepatitis, syphilis, and HIV. Serum lactate dehydrogenase was not detected. A pulmonary function test showed that his pulmonary ventilation function and diffusing capacity for carbon monoxide (DLco) were in normal reference ranges. Fractional exhaled nitric oxide concentration was 3.6 ppb.

**Fig. 1:**
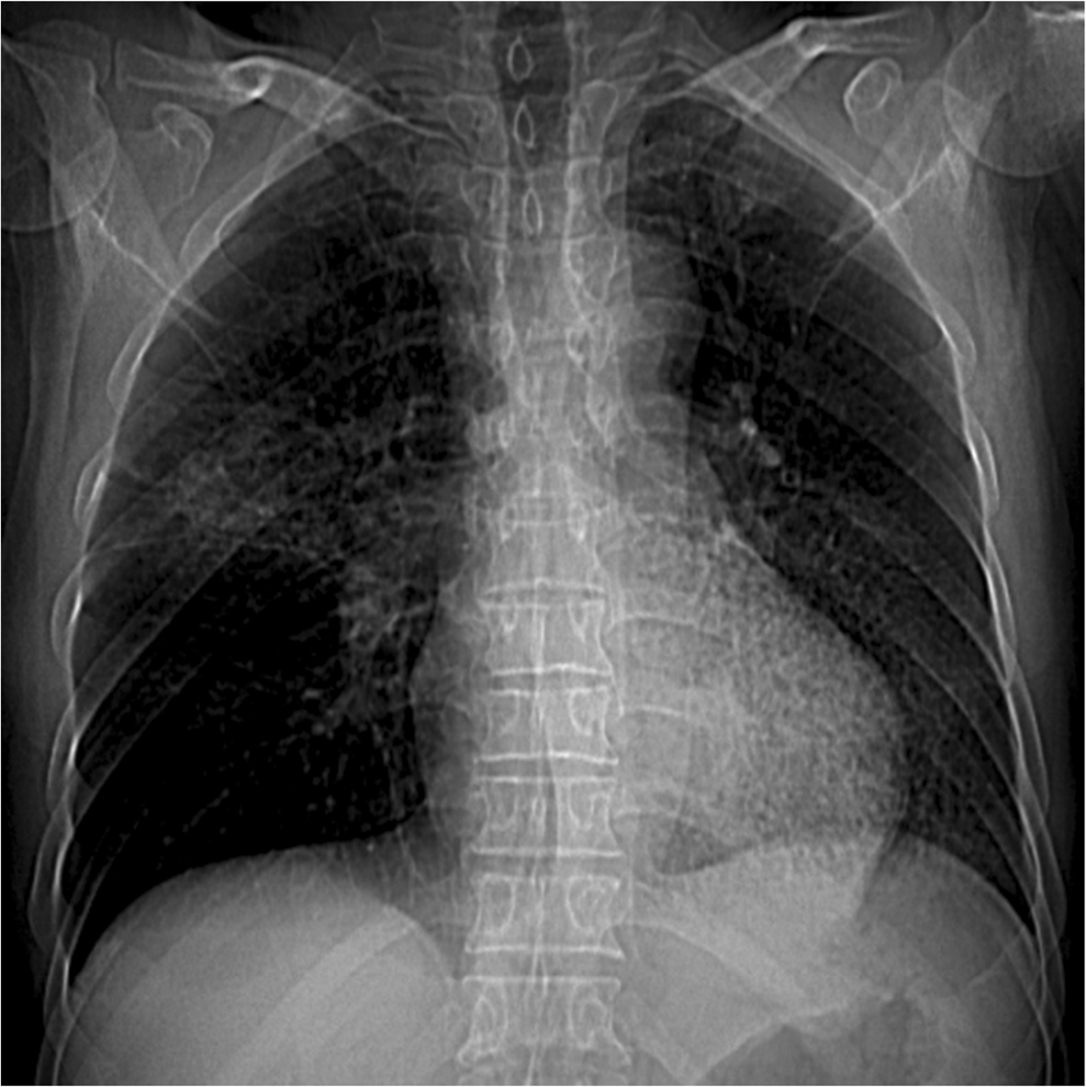
Chest imaging. Chest X-ray shows multiple bilateral nodules with surrounding bilateral reticular fibrotic lesions

**Fig. 2:**
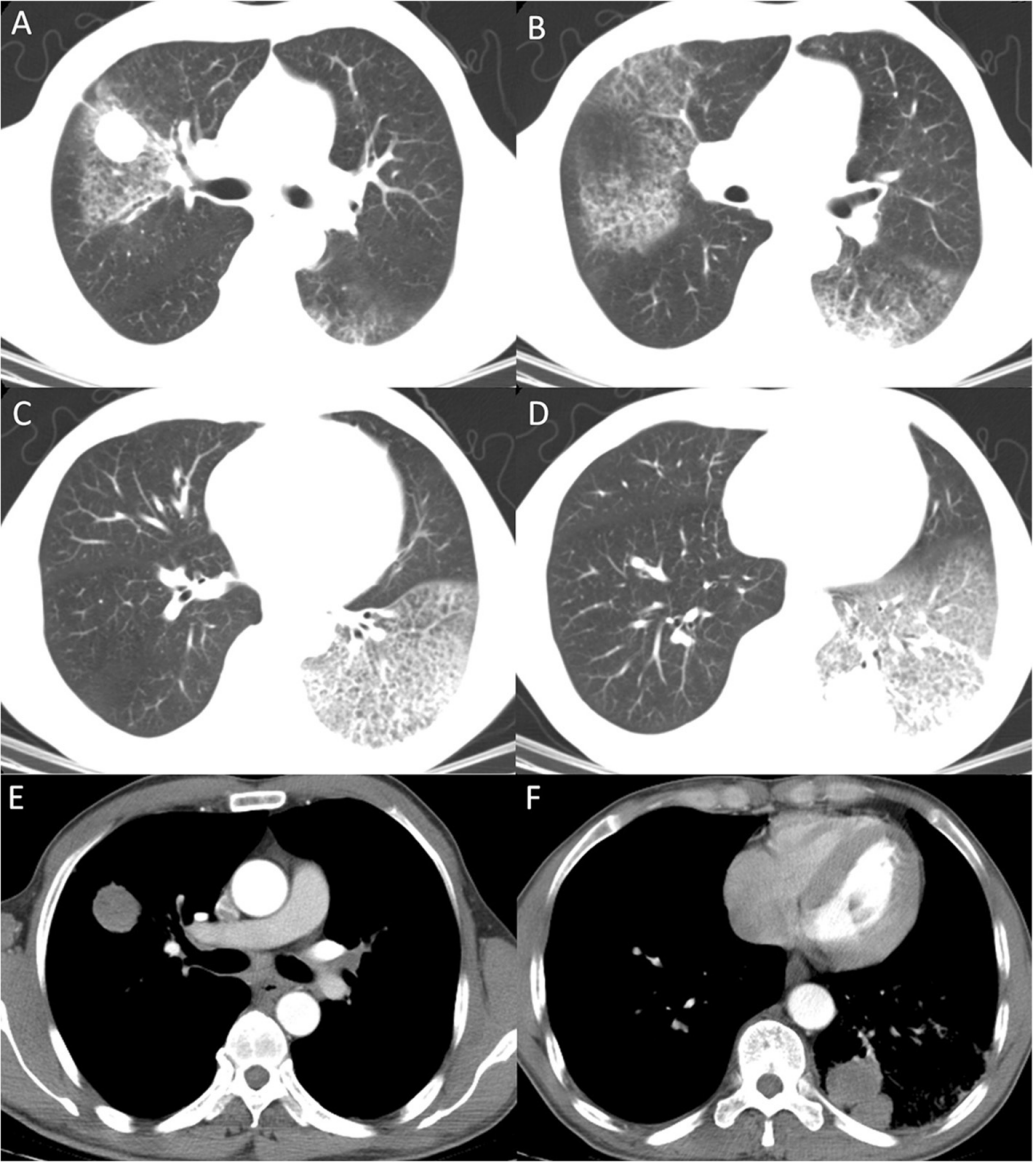
Sixty-year-old man with discrete bilateral nodules with surrounding “paving” pattern. Computed tomography (CT) enhancement scan shows discrete bilateral consolidations with a 28-mm × 24-mm nodule on the right upper lobe (**a**, **e**) and a 33-mm × 43-mm subpleural mass with slightly uneven enhancement on the left lower lobe (**d**, **f**), accompanied with intralobular interstitial thickenings shown as a surrounding bilateral paving pattern (**b**, **c**)

A postero-anterior chest radiograph showed bilateral nodules and bilateral fibrotic lesions (Fig. 1). An enhanced CT scan of the chest showed bilateral consolidations with a 28-mm × 24-mm nodule on the right upper lobe and a 33-mm × 43-mm subpleural nodular mass on the left lower lobe, accompanied with surrounding bilateral “paving” lesions (Fig. 2). Increased and enlarged lymph nodes in the right hilar, mediastinum, and left axillary fossa were noted. The CT scan also showed bilateral pleural thickening, fibrotic changes, and bilateral pleural effusions. The CT scan taken at the local hospital two months before admission to our department presented a similar imaging but with smaller nodules (A 27 mm * 24 mm nodule on the right upper lobe and a 30 mm* 21 mm mass on the left lower lobe) surrounded by “paving” lesions.

**Fig. 3:**
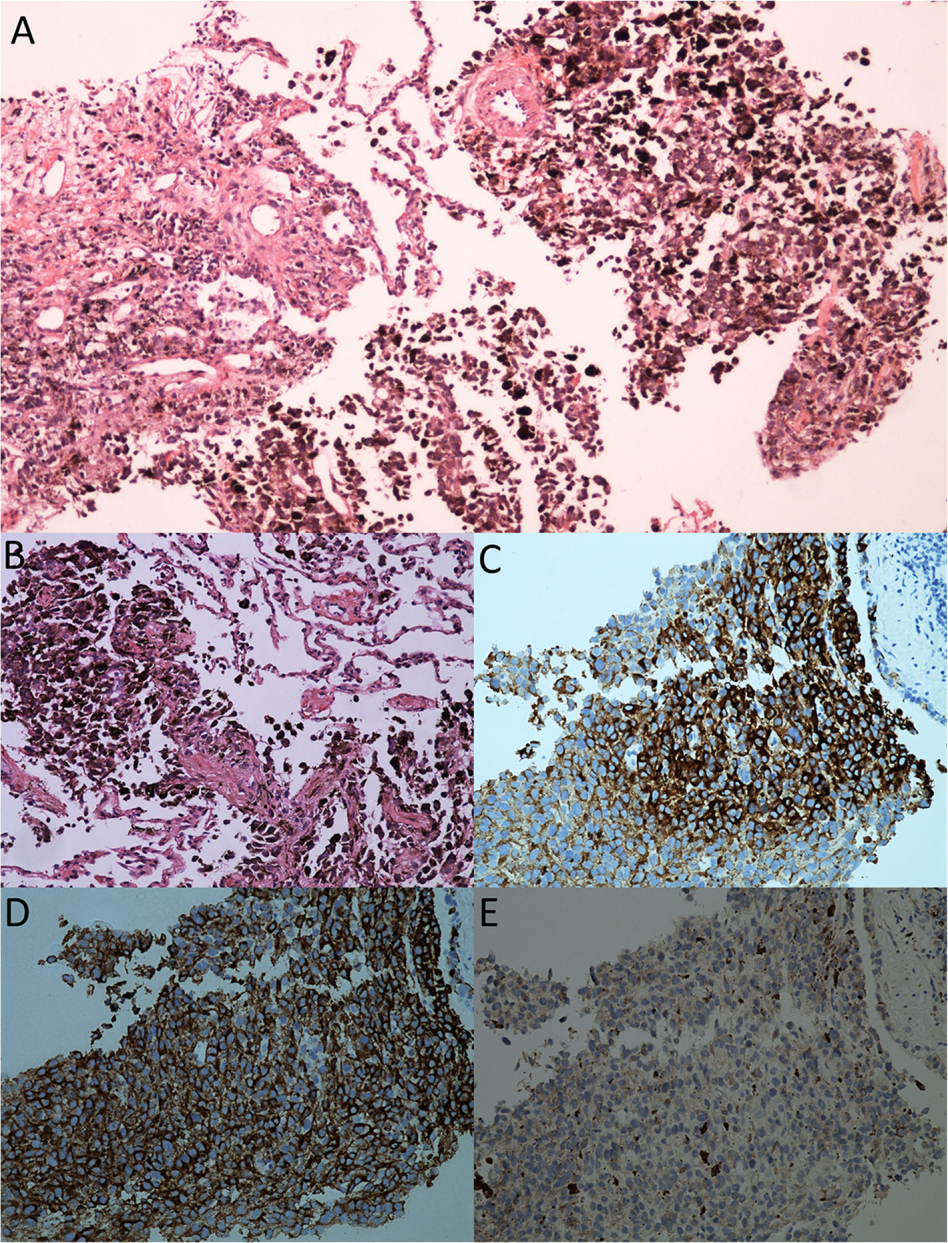
Immunohistochemical (IHC) findings of computed tomography-guided fine-needle aspiration. Histopathological examination of biopsy shows perivascular and intra-alveolar accumulated pigmented cells containing melanin granules (hematoxylin and eosin, ×100) (**a**). Histopathological features of intra-alveolar atypical cell accumulation accompanied with interstitial thickenings; pleomorphic cells with atypia are pigmented deep brown and were diagnosed as melanocytes (hematoxylin and eosin, ×200) (**b**). IHC staining for Melan-A is positive (original magnification × 200) (**c**). All tumor cell cytoplasm and focal nuclei show positive IHC staining for HMB-45 (original magnification × 200) (**d**). IHC staining for S-100 is negative (original magnification × 200) (**e**)

On presentation, the patient was treated with cefoperzone sodium/tazobactam and levofloxacin in case of pulmonary bacterial infection, which eventually showed no effectiveness. He then underwent a CT-guided fine needle aspiration biopsy from the left lung, which revealed pleomorphic cells with components of pigment granules. Immunohistochemical (IHC) staining was positive for human melanoma black-45 (HMB-45), Melan-A, and Ki-67 (LI 30 %), whereas staining for S-100 protein (Fig. 3), cytokeratin (CK5/6, CK7), CD68, CD56, P63, TTF-1, P40, Napsin A, ALK D5F3, ALK D5F3 N, Syn, and CgA were negative.

On establishing the diagnosis of melanoma, the patient refused chemotherapy or surgery for lack of money. He died two months later.

## Discussion

Although melanoma can invade all organs of the human body, the lung is the most common site of metastasis, and respiratory failure caused by metastatic lesions is the most common cause of death in patients with melanoma [3]. Melanomas in the lung are mostly metastatic; primary melanoma of the lung accounts for only 0.01 % of all primary pulmonary tumors [5]. Most patterns of pulmonary melanoma are solitary nodules, multiple nodules, and miliary peripheral pulmonary nodules; the miliary pattern implies a grave prognosis [4]. Few English-language reports of melanoma metastases to the lungs report nodules accompanied with diffuse infiltrates; fewer still describe any as discrete bilateral nodules with surrounding “paving” lesions.

Pulmonary lesions with paving patterns are usually diagnosed as pulmonary alveolar proteinosis (PAP). Although melanoma is rarely considered for such cases, paving patterns have been found in pulmonary melanoma. Shin et al. reported a diffuse infiltrative pattern consisting of intralobular interstitial thickenings and areas of ground-glass opacity in a pulmonary melanoma case, which was much like “paving” [6]. Kalchiem-Dekel et al. also presented a diffuse interstitial metastatic “paving” pattern in the lung in a 51-year-old male patient [7]. Although “paving” surrounding a mass has been found in pulmonary adenocarcinoma [8] and PAP [9], it has not been previously associated with melanoma, making our case the first description of invasive pulmonary melanoma manifested as bilateral discrete nodules with a surrounding “paving” pattern.

The patient had no history of excision of a cutaneous, mucosal, or ocular lesion, no evidence of suspicious pigmented lesions of the skin, mucosa, or eyes. He had no evidence of metastasis from any organs that can be examined. Even though, we could not calssify this case as a primary melanoma of lung as he could not fully meet the clinical criteria set forth by Jensen and Egedorf [10], especially when the patient had bilateral pulmonary lesions and did not went on an autopsy after death. What accords this case peculiarity is the pulmonary imaging pattern of melanoma, regardless of primary or metastatic involvement.

Another peculiarity of this case is that the biopsy was negative for S-100 protein in IHC staining. S-100 protein, along with HMB-45 and Melan-A, is a characteristic marker for melanocytes. Although melanoma is reportedly 83–100 % immunoreactive to S-100 protein [11], S-100 protein-negative melanoma has not been widely studied. Argenyi et al. re-evaluated 17 cases of melanomas that had previously tested negative for S-100 protein, and reassessed 8 of the 17 cases as positive for S-100 protein; 4 of the remaining S-100 protein-negative cases were positive for HMB-45 antigen, which is consistent with melanoma [11]. Although misdiagnoses may occur for technical reasons, some melanoma cases do not express S-100 protein at detectable levels. Lee et al. studied IHC patterns of five primary sinonasal melanomas and suggested that melanomas with small-cell morphology may be negative or only focally positive for S-100 protein [12]. Our case is the first presentation of S-100-negative pulmonary melanoma. Owing to the immunophenotypic heterogeneity of melanocytic lesions and the limitations of test technology, test results may require comprehensive evaluation, especially in cases of S-100-negative results for suspected melanoma.

The “paving” sign is characterized by a reticular pattern superimposed on ground-glass opacity in CT images. Pulmonary “paving” lesions are frequently diagnosed as PAP, but Lee Chang Hyun reviewed other possible causes of pulmonary “paving,” including Pneumocystis carinii pneumonia, bronchio-alveolar cell carcinoma, sarcoidosis, ARDS, pulmonary hemorrhage syndromes, acute radiation pneumonitis, and drug- induced pneumonitis [13].

Although many diseases can show this particular radiographic imaging style, the relationship between diseases and “paving” patterns has not been widely studied. To examine the associations between “paving” patterns and disease variety, we reviewed all English-language articles by searching MEDLINE (PubMed), EMBASE, and Web of Science for observational studies and case reports through December 2015. Searches were performed independently by two investigators on December 2015, using the following terms: “crazy paving,” “paving stone,” “paving stones,” “flagstone,” and “slabstone.”

We selected all studies that presented radiographic images of paving patterns. Any type of study design was considered, including case reports. We excluded duplicated reports and cases with pathologically unconfirmed or inaccurate clinical diagnoses. We also excluded literature with no cases based on radiographic images of paving patterns.

We identified 198 articles, of which 116 were excluded for not presenting any clinical radiographic images, and 1 was excluded for reporting the same cases in a different review. Finally, 81 citations were accepted for this review. Of these included studies, 29 were original clinical research, 1 was a review, and 51 were case reports. Collectively, they included 456 cases and, demonstrated more than 30 types of diseases—predominantly PAP (203/457, 44.42 %), viral pneumonia (85/457, 18.60 %), exogenous lipoid pneumonia (35/457, 7.66 %), pulmonary alveolar microlithiasis (12/457, 2.63 %), and bacterial pneumonia (28/457, 6.13 %; Table 1).

**Table 1:** Summary of reports of radiographic images of “paving” patterns and pathology results

Diseases	Case no. (pct.^a^)	References
Pulmonary alveolar proteinosis	203(44.42 %)	
Pulmonary alveolar proteinosis	24	Luo, J., et al. (2014) [16]
	35	Mehrian, P., et al. (2014) [9]
	14	Souza, C. A., et al. (2012) [17]
	15	Ishii, H., et al. (2009) [18]
	8	Choi, H. K., et al. (2010) [19]
	6	Spira, D., et al. (2013) [20]
	6	Cai, X., et al. (2005) [21]
	5	Coulier, B., et al. (1999) [22]
	5	Johkoh, T., et al. (1999) [14]
	3	Ishii, H., et al. (2009) [18]
	3	Mu, X. D., et al. (2008) [23]
	3	Akin, M. R. and G. K. Nguyen (2004) [24]
	2	Oh, S. J., et al. (2014) [25]
	2	Luo, J., et al. (2013) [26]
	1	Choi, Y. R., et al. (2015) [27]
	1	Oda, N., et al. (2015) [28]
	1	Kinehara, Y., et al. (2014) [29]
	1	Albores, J., et al. (2013) [30]
	1	Moisan, M., et al. (2013) [31]
	1	Langwieler, S., et al. (2012) [32]
	1	Jayaraman, S., et al. (2010) [33]
	1	Maimon, N. and D. Heimer (2010) [34]
	1	Ueda, Y., et al. (2009) [35]
	1	McDermott, H., et al. (2009) [36]
	1	Matsunaga, K., et al. (2008) [37]
	1	Sugimoto, C., et al. (2006) [38]
	1	De Arriba, C., et al. (2006) [39]
	1	Collard, B., et al. (2002) [40]
	1	Yokomura, K., et al. (2002) [41]
	1	Murayama, S., et al. (1999) [15]
Pediatric PAP	32	Enaud, L., et al. (2014) [42]
	22	Berteloot, L., et al. (2014) [43]
Children PAP	1	El-Dawlatly, A., et al. (2011) [44]
	1	Zontsich, T., et al. (1998) [45]
Exogenous lipoid pneumonia	35(7.66 %)	
	11	Marchiori, E., et al. (2010) [46]
	6	Choi, H. K., et al. (2010) [19]
	6	Lee, J. Y., et al. (1999) [47]
	5	Laurent, F., et al. (1999) [48]
	3	Zanetti, G., et al. (2007) [49]
	1	Nakashima, S., et al. (2015) [50]
	1	Schoofs, C., et al. (2010) [51]
	1	Sias, S. M., et al. (2009) [52]
	1	Meltzer, E., et al. (2006) [53]
Virus pneumonia	85(18.60 %)	
Influenza virus pneumonia	11	Kloth, C., et al. (2015) [54]
	8	Ono, A., et al. (2014) [55]
Influenza A (H1N1) pneumonia	11	Amorim, V. B., et al. (2013) [56]
	4	Henzler, T., et al. (2010) [57]
	1	Marchiori, E., et al. (2011) [58]
	1	Chandler, T. M., et al. (2010) [59]
Cytomegalovirus pneumonia	17	Kloth, C., et al. (2015) [54]
SARS-Coronovirus pneumonia	28	Wong, C. K., et al. (2012) [60]
Hantavirus pulmonary syndrome	1	Goncalves, F. G., et al. (2010) [61]
Human T-cell lymphotropic virus type 1 related pneumonia	3	Yamashiro, T., et al. (2012) [62]
Bacterial pneumonia	28(6.13 %)	
Bacterial pneumonia	7	Johkoh, T., et al. (1999) [14]
P. aeruginosa pneumonia	13	Kloth, C., et al. (2015) [54]
Streptococcus pneumoniae pneumonia	7	Ono, A., et al. (2014) [55]
	1	Ngo, M. H., et al. (2003) [63]
Pulmonary alveolar microlithiasis	12(2.63 %)	
	9	Francisco, F. A., et al. (2015) [64]
	1	McDermott, H., et al. (2009) [36]
	1	Roca Vanaclocha, Y., et al. (2008) [65]
	1	Gasparetto, E. L., et al. (2004) [66]
Pulmonary nocardiosis	5(1.09 %)	Tsujimoto, N., et al. (2012) [67]
Granulomatous mycosis fungoides	1(0.22 %)	Sverzellati, N., et al. (2006) [68]
Tuberculosis	1(0.22 %)	Huang, H. and P. X. Lu (2013) [69]
	1(0.22 %)	Johkoh, T., et al. (1999) [14]
Mycoplasma pneumonia	2(0.44 %)	Johkoh, T., et al. (1999) [14]
*P carinii*-induced pneumonia	4(0.88 %)	Murayama, S., et al. (1999) [15]
	1(0.22 %)	Johkoh, T., et al. (1999) [14]
Pulmonary Leptospirosis	3(0.66 %)	von Ranke, F. M., et al. (2015) [70]
	1(0.22 %)	Marchiori, E., et al. (2008) [71]
Obstructive pneumonitis	1(0.22 %)	Johkoh, T., et al. (1999) [14]
ARDS	8(1.75 %)	Johkoh, T., et al. (1999) [14]
	1(0.22 %)	Murayama, S., et al. (1999) [15]
Pulmonary hemorrhage	3(0.66 %)	Johkoh, T., et al. (1999) [14]
	1(0.22 %)	Murayama, S., et al. (1999) [15]
Chronic eosinophilic pneumonia	2(0.44 %)	Johkoh, T., et al. (1999) [14]
Hypersensitivity pneumonitis	1(0.22 %)	Scordino, D. and L. Regan (2014) [72]
Interstitial pneumonia	1(0.22 %)	Chen, G. L., et al. (2014) [73]
Usual interstitial pneumonia	2(0.44 %)	Johkoh, T., et al. (1999) [14]
	1(0.22 %)	Murayama, S., et al. (1999) [15]
Non-specific interstitial pneumonia	1(0.22 %)	Coche, E., et al. (2001) [74]
Acute interstitial pneumonia	5(1.09 %)	Johkoh, T., et al. (1999) [14]
Organizing pneumonia	1(0.22 %)	Utrilla Contreras, C., et al. (2014) [75]
Bronchiolitis obliterans organizing pneumonia	1(0.22 %)	Johkoh, T., et al. (1999) [14]
Systemic lupus erythematosus	1(0.22 %)	Hisada, T., et al. (2006) [76]
Nonclassifiable interstitial pneumonia	4(0.88 %)	Song, I., et al. (2012) [77]
Malignant melanoma metastasis	1(0.22 %)	Kalchiem-Dekel, O., et al. (2015) [7]
Peripheral T-cell lymphoma	1(0.22 %)	Fraser, T. and A. Nagarur (2015) [78]
Adult T-cell leukemia or lymphoma	4(0.88 %)	Okada, F., et al. (2004) [79]
AIDS-related Kaposi sarcoma	1(0.22 %)	Matsunaga, K., et al. (2008) [37]
Pulmonary lymphedema	1(0.22 %)	Ohnishi, H., et al. (2014) [80]
Cardiogenic pulmonary edema	2(0.44 %)	Johkoh, T., et al. (1999) [14]
	1(0.22 %)	Senturk, A., et al. (2013) [81]
	1(0.22 %)	Maimon, N., et al. (2006) [82]
Niemann-Pick disease	1(0.22 %)	Castanon Martinez, R., et al. (2012) [83]
Idiopathic pneumonia syndrome after bone marrow transplantation	1(0.22 %)	Gasparetto, T. D., et al. (2008) [84]
Barium aspiration	1(0.22 %)	Akata, S., et al. (2007) [85]
Bronchioloalveolar cell carcinoma	2(0.44 %)	Que, C. L., et al. (2006) [86]
	1(0.22 %)	Kunimasa, K., et al. (2015) [87]
	1(0.22 %)	Rossi, S. E., et al. (2003) [8]
Noncardiogenic pulmonary edema	1(0.22 %)	Takeshita, T., et al. (2005) [88]
Squalene aspiration pneumonia	8(1.75 %)	Lee, K. H., et al. (2005) [89]
Thoracic lymphangiectasis	1(0.22 %)	Orliaguet, O., et al. (2002) [90]
Near drowning	3(0.66 %)	Kim, K. I., et al. (2000) [91]
Radiation pneumonitis	3(0.66 %)	Johkoh, T., et al. (1999) [14]
	1(0.22 %)	Murayama, S., et al. (1999) [15]
Drug-induced pneumonitis	3(0.66 %)	Johkoh, T., et al. (1999) [14]
	1(0.22 %)	Murayama, S., et al. (1999) [15]
Total	457	

^a^Percentage of reported cases among all cases reviewed above

Most of the 29 original studies were retrospective comparisons of radiographic appearance of different diseases; few reported on “paving” as a sign of different diseases. Johkoh et al. investigated the spectrum of disease associated with “crazy paving,” and found 46 patients with 15 identified diseases, including ARDS (8/46, 17.4 %), bacterial pneumonia (7/46, 15.2 %), acute interstitial pneumonia (5/46, 10.9 %), PAP (5/46, 10.9 %), radiation pneumonitis (3/46, 6.5 %), drug-induced pneumonitis (3/46, 6.5 %), pulmonary hemorrhage (2/46, 4.3 %), chronic eosinophilic pneumonia (2/46, 4.3 %), cardiogenic pulmonary edema (2/46, 4.3 %), usual interstitial pneumonia (2/46, 4.3 %), mycoplasma pneumonia (2/46, 4.3 %), as well as tuberculosis (1/46, 2.2 %), obstructive pneumonitis (1/46, 2.2 %), *P carinii*-induced pneumonia (1/46, 2.2 %), and bronchiolitis obliterans organizing pneumonia (1/46, 2.2 %) [14]. Murayama et al. also reviewed 10 patients, including those with P carinii-induced pneumonia and ARDS, pulmonary hemorrhage, radiation pneumonitis, drug-induced pneumonitis, PAP, and usual interstitial pneumonia showing pulmonary “paving,” with P carinii- induced pneumonia being the most common [15].

Pulmonary alveolar proteinosis (including cases found in adults, children, and infants) accounted for 44.42 % (203/457) of all cases we found in the literature. It was by far the most common presentation, followed by viral pneumonia (85/457, 18.60 %), exogenous lipoid pneumonia (35/457, 7.66 %), bacterial pneumonia (28/457, 6.13 %), and pulmonary alveolar microlithiasis (12/457, 2.63 %). Among the viral pneumonia cases, influenza virus was the most common pathogen (36 patients), followed by SARS-coronavirus (28 patients), cytomegalovirus (17 patients), human T-cell lymphotropic virus type 1 (3 patients), and Hantavirus (1 patient). Although these percentages cannot show precise incidence rates for each disease that can show “paving,” they may offer clues to causation.

According to the studies we reviewed (Table 1), more than 40 diseases can reportedly show paving patterns in lung images, including pulmonary nocardiosis, granulomatous mycosis fungoides, pulmonary leptospirosis, hypersensitivity pneumonitis, non-specific interstitial pneumonia, organizing pneumonia, systemic lupus erythematosus, non-classifiable interstitial pneumonia, lymphoma, leukemia, AIDS-related Kaposi sarcoma, pulmonary lymphedema, Niemann–Pick disease, idiopathic pneumonia syndrome after bone marrow transplantation, barium aspiration, squalene aspiration pneumonia, bronchiolo-alveolar cell carcinoma, noncardiogenic pulmonary edema, thoracic lymphangiectasis, and near drowning, in addition to the diseases mentioned above.

## Conclusions

Here, we describe the first case of pulmonary melanoma in the form of discrete bilateral nodules with a paving pattern, although it is not the first case of pulmonary melanoma with a “crazy paving” imaging, making melanoma another of more than 40 diseases that can appear as paving patterns in chest images. Although paving patterns are rare, physicians should consider pulmonary melanoma in differential diagnoses of patients who display this sign.

## Abbreviations

CT, computed tomography; PAP, pulmonary alveolar proteinosis
